# Preoperative prediction model for risk of readmission after total joint replacement surgery: a random forest approach leveraging NLP and unfairness mitigation for improved patient care and cost-effectiveness

**DOI:** 10.1186/s13018-024-04774-0

**Published:** 2024-05-10

**Authors:** Varun Digumarthi, Tapan Amin, Samuel Kanu, Joshua Mathew, Bryan Edwards, Lisa A Peterson, Matthew E Lundy, Karen E Hegarty

**Affiliations:** 1 Novant Health Cognitive Computing, Novant Health, Inc, Winston-Salem, NC USA; 2 Novant Health Presbyterian Medical Center, Novant Health, Inc, Charlotte, NC USA

**Keywords:** Predictive model, Classification, Fairlearn, Natural language processing, Orthopedic

## Abstract

**Background:**

The Center for Medicare and Medicaid Services (CMS) imposes payment penalties for readmissions following total joint replacement surgeries. This study focuses on total hip, knee, and shoulder arthroplasty procedures as they account for most joint replacement surgeries. Apart from being a burden to healthcare systems, readmissions are also troublesome for patients. There are several studies which only utilized structured data from Electronic Health Records (EHR) without considering any gender and payor bias adjustments.

**Methods:**

For this study, dataset of 38,581 total knee, hip, and shoulder replacement surgeries performed from 2015 to 2021 at Novant Health was gathered. This data was used to train a random forest machine learning model to predict the combined endpoint of emergency department (ED) visit or unplanned readmissions within 30 days of discharge or discharge to Skilled Nursing Facility (SNF) following the surgery. 98 features of laboratory results, diagnoses, vitals, medications, and utilization history were extracted. A natural language processing (NLP) model finetuned from Clinical BERT was used to generate an NLP risk score feature for each patient based on their clinical notes. To address societal biases, a feature bias analysis was performed in conjunction with propensity score matching. A threshold optimization algorithm from the Fairlearn toolkit was used to mitigate gender and payor biases to promote fairness in predictions.

**Results:**

The model achieved an Area Under the Receiver Operating characteristic Curve (AUROC) of 0.738 (95% confidence interval, 0.724 to 0.754) and an Area Under the Precision-Recall Curve (AUPRC) of 0.406 (95% confidence interval, 0.384 to 0.433). Considering an outcome prevalence of 16%, these metrics indicate the model’s ability to accurately discriminate between readmission and non-readmission cases within the context of total arthroplasty surgeries while adjusting patient scores in the model to mitigate bias based on patient gender and payor.

**Conclusion:**

This work culminated in a model that identifies the most predictive and protective features associated with the combined endpoint. This model serves as a tool to empower healthcare providers to proactively intervene based on these influential factors without introducing bias towards protected patient classes, effectively mitigating the risk of negative outcomes and ultimately improving quality of care regardless of socioeconomic factors.

## Background

The Centers for Medicare and Medicaid Services (CMS) is a US federal agency that manages Medicare and Medicaid healthcare programs. CMS has played a vital role in addressing readmissions, in accordance with the initiatives stemming from the Affordable Care Act. One of the implementations of such initiatives is the development of the Hospital Readmissions Reduction Program (HRRP) which enforces financial penalties on hospitals with high rates of avoidable readmissions. These penalties on readmission rates are focused on certain conditions, including pneumonia, acute myocardial infarction, and heart failure. This list was later revised in 2014 to include elective primary Total Hip Arthroplasty (THA) and Total Knee Arthroplasty (TKA) surgeries. In addition to the negative effect readmission has on a patient’s well-being, it is also a significant financial concern that increases healthcare costs. As a result, healthcare policymakers prioritized reducing avoidable readmissions. Overall, the HRRP has motivated hospitals to re-evaluate and optimize their care transitions, care coordination, and interventions to prevent avoidable readmissions. The aging boomer population and associated rise in degenerative joint diseases are expected to increase the demand for Total Joint Arthroplasty (TJA) surgeries in the upcoming years. An astonishing 1.25 million hip and knee arthroplasty surgeries were performed in 2019 alone (https://datatools.ahrq.gov/hcup-fast-stats). Moreover, a study conducted in 2019 forecasted an anticipated increase to 3.42 million total knee arthroplasty (TKA) surgeries by 2040 and to 1.43 million for THA surgeries [1] Likewise, a 2020 study employed projection models to predict a remarkable 235.2% surge in total shoulder arthroplasty (TSA) surgeries from 2017 to 2025, estimating that TSA procedures would rise to 350,558 annually by 2025 [2] While TSA surgeries are not included in the HRRP program, TSA surgeries were included in this model due to their dramatic rising occurrence and similarity in underlying drivers of readmission to lower joint arthroplasty. This increase in demand carries a risk of increasing readmissions and thus their associated penalties and costs.

Though the HRRP is a Medicare construct, all patients were considered in this study regardless of medical coverage provider. While the overall goal is to reduce readmission rates to avoid CMS financial penalties, we posit that the benefit of a predictive model can be extended beyond Medicare patients. Thus, in harmony with the healthcare philosophy of providing equitable care, our model does not exclude any patients based on their payor status.

Emergency Department (ED) visits within 30 days of surgery are also being considered as part of a combined endpoint in this study as private insurance providers are concerned not only with readmissions but also with ED visits following the surgery. When investigating why and when patients are being discharged to a Skilled Nursing Facility (SNF) or Rehabilitation facility (Rehab), it is evident that this decision is being made by care teams after the surgery while considering several risk factors of a readmission. Patients who are discharged to a SNF / Rehab facility following the surgery are considered as part of the combined endpoint because from a predictive and clinical point of view, the objective is to identify higher risk patients that will need additional clinical intervention to prevent readmission and ensure a smooth recovery.

Similar studies have been conducted in the past to better understand the risk factors associated with 30-day readmissions [3, 4]. This work brings two significant advancements the authors have not identified in prior work. First, a Natural Language Processing (NLP) algorithm was implemented to digest unstructured text from communications between patients and providers and in clinical progress notes made throughout the course of care. The application of NLP enables the valuable information locked within that unstructured text to inform the projected risk. Second, a simple yet elegant improvement was made to a prior methodology of threshold optimization from the Fairlearn package (https://fairlearn.org/) for mitigation of gender and payor bias in model performance and selection [5]. This study was driven by our care team to identify high risk patients prior to their surgery via an automatic and data-driven mechanism to comprehensively review the patient’s clinical history and potential risk factors. Model transparency is achieved by providing the top three risk factors contributing to increasing an individual patient’s risk to the patient’s pre-surgery care team, thus facilitating a data driven intervention plan for each patient prior to their surgery.

This research is an in-depth exploratory study which showcases the integration of multiple machine learning techniques to build a robust predictive model. By leveraging sophisticated methodologies like unfairness mitigation, propensity score matching, feature selection, and natural language processing, the model can predict the risk of endpoint before surgery occurs, thereby equipping healthcare practitioners with a powerful tool with model explainability both at the global and patient level. This model serves as an early risk assessment tool in the pre-surgery window to help facilitate clinical decision making with proactive interventions, thereby reducing healthcare costs associated with the outcomes.

## Methods

### Data collection

This study adhered to a protocol approved by the Novant Health Institutional Review Board (IRB) and followed the ethical guidelines outlined in the Helsinki Declaration of 1975. A consent waiver was obtained due to the minimal risk of harm involved in this research, ensuring that subjects’ rights and welfare were not compromised and that the research could not be practically carried out without the waiver.

The data for the study was obtained from existing EHR data housed in Novant Health’s Epic (https://www.epic.com/) Clarity database. Novant Health is a not-for-profit health system situated in the southeastern United States. The health system is comprised of 16 medical centers with 3,333 licensed beds, 176 operating rooms and a medical group consisting of over 1,922 physicians practicing at more than 800 ambulatory clinics. The dataset included 32,405 patients who underwent total knee, hip, or shoulder replacement surgeries between January 1, 2015, and December 31, 2021. In total 38,581 surgical cases were gathered, accounting for the fact that a patient can have more than one surgical case.

Cases of elective total hip, knee, and shoulder arthroplasty surgeries were identified with the help of International Classification of Diseases (ICD) and Current Procedural Terminology (CPT) codes. Cases were excluded from the study if the patient had pre-existing fractures, as this would be considered non-elective, or underwent surgeries other than total replacement such as revisions, resurfacing, repairs, and partial replacement. Cases with patient fatalities during the surgery were not included in this study.

### Combined endpoint creation

A case was labelled as positive if they had at least one of the following:

a) An inpatient admission within 30 days from their discharge date,

b) An ER admission within 30 days from their discharge date, or.

c) A discharge disposition from surgery to a SNF / Rehab facility.

If the patient was already in a SNF and then discharged to a SNF following surgery, the case would not be considered as a readmission. Cases with planned readmissions related to specific diagnoses such as pregnancy/delivery, breast implantation, artificial/prosthetic limbs and procedure groupers such as organ transplantation were also excluded from the dataset in this study.

### Feature extraction

A broad set of 98 potential features which included history of laboratory results, diagnoses, vitals, medications, utilizations, social history and demographics were initially obtained for the patient population. To create diagnosis features, patient’s individual diagnoses are grouped based on Clinical Classifications Software categories (https://www.nlm.nih.gov). Diagnosis features were extracted from various sources such as encounter diagnoses, problem list, diagnoses information from claims and diagnoses associated with surgeries. All diagnoses features are binary, indicating whether the diagnosis has been noted within 1 year prior to when the surgical case was created. Medications were sourced from the patient’s active medications list within 90 days prior to surgical case creation date. Medication features were created based on the medication’s pharmaceutical class. Social history and demographic features include age at the time of surgical case creation, whether the patient is female versus all other genders, whether the patient is currently using any tobacco products, whether the patient drinks alcohol, whether the patient uses any illegal substances, whether the patient has a significant other, whether the patient has a primary care provider, and whether the patient is enrolled in Medicare and/or Medicaid versus all other payers. To generate the lab features, a patient’s lab results within the past two years of surgical case creation date were extracted. Utilization features are features that give a better understanding of a patient’s visitation activity across various venues of care throughout the health system. Utilization features include the number of ED visits the patient has had in the two years prior to surgical case creation date, the number of in-patient admissions the patient has had in the year prior to the surgical case creation date, the number of no-show appointments the patient has had in the 90 days prior to surgical case creation date, the number of cancelled appointments the patient has had in the 90 days prior to surgical case creation date, the number of office visits the patient has had in the 90 days prior to surgical case creation date, the maximum length of stay the patient had in the two years prior to surgical case creation date, whether the patient had an ED visit with a chief complaint of abdominal pain in the two years prior to surgical case creation date, whether patient had an ED visit with a chief complaint of chest pain in the two years prior to surgical case creation date, and whether patient had an ED visit with a chief complaint of shortness of breath in the two years prior to the surgical case creation date.

In addition to the above-mentioned features, whether the patient had a fall history within the last year, the patient’s clinically estimated risk of fall, and Charlson Co-morbidity index score were included [6]. The Charlson score is a method of quantifying comorbidities of patients based on the ICD diagnosis codes. Each comorbidity category has an associated weight (from 1 to 6), and the sum of all the weights results in a single comorbidity score for a patient. A score of zero indicates that no comorbidities were found. Most recent BMI value prior to surgical case creation date was also included as a feature.

### NLP feature extraction

Clinical text such as progress notes, discharge summary, nursing notes, and ED notes for each patient were extracted from the patient’s EHR in the 120 days prior to surgical case creation date. To maintain data integrity, duplicate notes were identified and removed. Clinical notes were then aggregated at the level of surgical cases, ensuring that they were placed in descending order, with the most recent notes being positioned at the beginning. Out of 38,581 cases, 29,346 cases had clinical notes populated. On an average each case had around 23 clinical notes. As part of the data pre-processing, notes with less than 200 characters were filtered out. Additionally, leading and trailing spaces were replaced by a single space. Consecutive repetition of special characters such as “=”, “_”, “-” were removed from the notes. After data cleaning, the average length of notes for a case was 37,237 characters.

BioClinicalBERT is a tailored version of the BERT (Bidirectional Encoder Representations from Transformers) model that has been initialized from BioBERT and trained on clinical notes from MIMIC III [7, 8]. By leveraging transfer learning, BioClinicalBERT was fine-tuned on the downstream task of sentence classification of the patient’s clinical notes in this study. The BioClinicalBERT model was fine-tuned using Hugging Face (https://huggingface.co/) on four GPU-accelerated standard NC24s with NVIDIA Tesla V100 GPUs. During the fine-tuning process, the initial step was to prepare the inputs for the model using a pre-trained tokenizer of BioClinicalBERT. Tokenization involves breaking up strings from the notes of each case into sub-word token strings and converting them into specific IDs determined by the pre-trained BioClinicalBERT tokenizer. Each input in the tokenized encodings has a maximum length of 512. Each input starts with the token “101” indicating it is for classification and ends with “102” indicating the end of input. If the input is less than length 512, then rest of the ids were padded with “0”. Each input was then associated with its combined prediction endpoint. Pretrained model weights and configurations were downloaded using Hugging Face. With the AdamW optimizer, the model was trained for 4 epochs (fine-tuned) using a learning rate of 3 × 10^− 6^ and a batch size of 8 on the tokenized notes data to classify its associated combined endpoint label. The initial training time was around eight hours. Mixed precision was used to substantially reduce the NLP model training time by nearly 40%. This technique performs as many operations as possible in half-precision floating point, fp16, instead of the default single-precision floating point, fp32. Mixed precision uses different precision levels within a single operation to achieve computational efficiency without sacrificing accuracy. Training time was reduced to five hours without loss in performance by using mixed precision. Hyperparameters were explored as follows: learning rate {3 × 10^− 6^, 3 × 10^− 7^, 2 × 10^− 6^, 4 × 10^− 6^}, epochs {2, 3, 4, 5}, maximum sequence length {128, 256, 512}.

Because each input is associated with its combined endpoint and each surgical case may have multiple text inputs, the model produces multiple predictions for each surgical case. The predictions for each case were combined using the below formula [9]:

$${P}_{final} =\frac{{P}_{max}+{P}_{mean }\cdot \frac{n}{C}}{1+\frac{n}{C}}$$

In the above formula, *n* represents the number of subsequences which is the number of chunks a surgical case’s note text was broken down into. The scaling factor *C* controls the influence of *n*. The maximum and average probabilities of readmission over n subsequences are *P*_*max*_ and *P*_*mean*_ respectively. C varied over the range 1 to 100 in increments of 0.5 with the objective of optimizing the F1 score of the model. The C value of 92.5 had the best performance. The final probability *P*_*final*_ (NLP risk score) indicates the risk of combined endpoint derived from the patient’s notes and was added as an additional feature.

### Descriptive statistics

The total number of surgical cases by type of joint are shown in Table 1 along with their breakdown by combined endpoint. The prevalence of the combined endpoint in this study was around 16%. It can also be observed that more than 50% of surgeries were TKA. The average age of this study’s population was around 66 years with a standard deviation (SD) of 10, indicating that the elderly population is more likely to get these joint replacement surgeries. Mean BMI (SD) was around 31.6 [6] putting the average population member in the overweight category. Descriptive information for all continuous features is shown in Table 2. Around 59% of the population were females and 60% of the population had active Medicare coverage. Also, among the population, 54% were diagnosed with connective tissue conditions and 51% diagnosed with hypertension. The Kendall Tau (Kendall Rank Correlation Coefficient) was used to find the correlation between the 28 continuous features. Kendall’s Tau is a non-parametric measure of relationship correlation measure of relationships between columns of ranked data. The Tau correlation coefficient returns between − 1 and + 1 where + 1 indicates that all pairs are concordant, -1 indicates that all pairs are discordant and a value of 0 indicates no relation [10]. The only features found to have a high positive correlation with each other were RBC, HCT, hemoglobin and maximum stay length, inpatient admissions with values above 0.65. The Cramér’s V measure was used to find the correlation between the 70 categorical/binary features. Cramér’s V is a measure of association between two nominal variables, giving a value between 0 and 1 where 0 indicates no association and 1 indicates a perfect association between the two variables [11]. No high correlations were observed between the categorical features.

**Table 1:** Surgeries considered in study, by joint type and endpoint

Surgery type	Number of surgeries (% of total population)	Number having combined endpoint labeled positive (% of surgery type population)	Number having combined endpoint labeled negative (% of surgery type population)
Knee	21,903 (56.8%)	4025 (18.4%)	17,878 (81.6%)
Hip	13,021 (33.7%)	1938 (14.9%)	11,083 (85.1%)
Shoulder	3657 (9.5%)	457 (12.5%)	3200 (87.5%)
Total	38,581 (100%)	6420 (16.6%)	32,161 (83.4%)

**Table 2:** Statistics for continuous features

Predictor	Mean (SD)	Min / 25% / 50% / 75% / Max
Age	66.54 (10.09)	18.06 / 59.91 / 67.15 / 73.65 / 98.92
BMI	31.68 (6.06)	13.65 / 27.25 / 31.17 / 35.73 / 70.49
Number of ED visits	0.53 (1.49)	0.0 / 0.0 / 0.0 / 1.0 / 65.0
Max stay length	0.71 (2.14)	0.0 / 0.0 / 0.0 / 0.0 / 59.0
A1C	6.0 (0.92)	4.1 / 5.5 / 5.8 / 6.3 / 15.1
Charlson score	2.06 (2.54)	0.0 / 0.0 / 1.0 / 3.0 / 21.0
Number of no show appts	0.18 (0.59)	0.0 / 0.0 / 0.0 / 0.0 / 28.0
Prothrombin time	13.46 (5.0)	8.4 / 10.6 / 12.4 / 14.2 / 88.9
Number of office visits	1.56 (1.81)	0.0 / 0.0 / 1.0 / 2.0 / 25.0
Total bilirubin	0.5 (0.28)	0.1 / 0.3 / 0.4 / 0.6 / 5.9
Number of cancelled appts	1.08 (2.07)	0.0 / 0.0 / 0.0 / 1.0 / 62.0
Albumin	4.25 (0.35)	1.5 / 4.1 / 4.3 / 4.5 / 5.5
Blood urea nitrogen	16.48 (6.33)	2.0 / 12.0 / 16.0 / 19.0 / 103.0
Total protein	6.88 (0.5)	3.8 / 6.6 / 6.9 / 7.2 / 10.0
Alanine transaminase	21.68 (15.72)	3.0 / 14.0 / 18.0 / 25.0 / 650.0
Fall risk	44.09 (27.88)	0.0 / 18.0 / 44.75 / 67.2 / 100.0
Alkaline phosphate	82.05 (31.48)	12.0 / 64.0 / 78.0 / 95.0 / 1622.0
Hemoglobin	13.17 (1.77)	6.0 / 12.1 / 13.3 / 14.4 / 20.3
Creatinine	0.92 (0.39)	0.2 / 0.73 / 0.86 / 1.03 / 15.1
White blood cell	7.69 (2.87)	0.7 / 5.8 / 7.1 / 9.0 / 71.6
Red blood cell	4.42 (0.58)	1.98 / 4.07 / 4.45 / 4.79 / 7.43
Hematocrit	39.75 (4.9)	18.3 / 36.9 / 40.1 / 43.0 / 62.1
Platelets	248.71 (70.52)	29.0 / 201.0 / 242.0 / 287.0 / 993.0
NLP risk score	0.38 (0.19)	0.03 / 0.22 / 0.35 / 0.52 / 0.9
Number of inpatient admissions	0.18 (0.49)	0.0 / 0.0 / 0.0 / 0.0 / 17.0
Partial thromboplastin time	30.14 (8.66)	17.0 / 26.0 / 28.0 / 32.0 / 142.2
Number of surgeries	0.36 (0.7)	0.0 / 0.0 / 0.0 / 1.0 / 15.0
Protein in urine	35.17 (50.73)	3.0 / 7.2 / 16.3 / 35.5 / 282.1

### Feature selection

During feature selection, features with significant levels of missing values (above 70% null values) such as protein urine content, total protein, fall history, fall risk, prothrombin time, and partial prothrombin time were excluded. A combination of Cook’s distance and standardized residuals was used to remove any strong influential outliers from the data. Cook’s distance is a measure of the influence of an observation in least squares regression analysis [12]. Removing any observations with a large Cook’s distance will greatly impact fitted coefficients in a regression model. Standardized residuals are defined for each observation as an ordinary residual divided by an estimate of its standard deviation and if that value is larger than three, it is considered an outlier. A total of 422 identified outliers were removed during the feature selection process. Thus, 38,159 cases were used for feature selection process. Multicollinear features were identified based on their Variance Inflation Factor (VIF). VIF measures how much the variance of an independent variable is influenced, or inflated, by its interaction/correlation with the other independent variables. Variables with a variance inflation factor of one are not correlated, values between 1 and 5 imply moderate correlation and anything greater than five is highly correlated. As shown in the Table 3, the hematocrit (HCT) and hemoglobin features had high VIF (> 5) indicating high multicollinearity. Thus, those two features were dropped during the feature selection process. Categorical features with null values were given a separate category of “unknown” and continuous features with null values were imputed using Random Forest Regression models considering non-linear relationship between features. Each feature with missing values was considered as a target variable for the model and all other features without missing values were used as predictor variables to train the model and impute missing values with predictions made by the model.

**Table 3:** Variance inflation factor (VIF)

Predictor	VIF	VIF after removing hematocrit and hemoglobin
Alkaline phosphate	1.05	1.05
Alanine transaminase	1.08	1.07
A1C	1.09	1.08
White blood cell	1.11	1.11
BMI	1.12	1.11
Total bilirubin	1.12	1.09
Platelets	1.13	1.10
Number of no show appts	1.18	1.18
Albumin	1.19	1.17
Number of ED visits	1.29	1.29
Charlson score	1.31	1.31
Age	1.32	1.31
Number of cancelled appts	1.34	1.34
Number of office visits	1.37	1.37
NLP risk score	1.38	1.38
Number of surgeries	1.39	1.39
Creatinine	1.41	1.41
Blood urea nitrogen	1.47	1.47
Max stay length	1.57	1.57
Number of inpatient admissions	1.94	1.92
Red blood cell	4.47	1.24
Hemoglobin	15.44	-
Hematocrit	18.68	-

Using the Box-Tidwell test, the linear relationship between explanatory variables and the logit of the response variable in the imputed data set was checked. This is achieved by including log-transformed interaction terms between the continuous explanatory variables and their corresponding natural log into the model. Features such as Number of Inpatient Admissions, Charlson Score, Patient Office Visits, Maximum Length of Stay, Alanine Transaminase Content, BMI, Alkaline Phosphatase, Age, Creatinine level, and White Blood Cells count indicated non-linearity from the test based on the statistical significance (*p* ≤ 0.05) using p-values. Thus, the Box-Cox transformation was applied to these features. The Box Cox transformation is a statistical technique which transforms the data more closely to a normal distribution. An Adaptive Lasso approach was used to select the final set of features. Adaptive Lasso is a regularization method involving an initial run of ridge regression to obtain coefficients used as weights for the lasso regression. Adaptive Lasso was selected because of its oracle property and thus has a consistent variable selection [13]. Different rates of regularizations were applied for each coefficient based on their weights. This ensured that the Adaptive Lasso penalized the coefficients with lower initial estimates more. The formula below was utilized for calculating the weights which work as a penalty factor for each individual feature in the lasso regression.

$$\widehat{{\omega }_{j}}=\frac{1}{{\left(\left|{\widehat{\beta }}_{j}^{ini}\right|\right)}^{\gamma }}$$

In the equation above, ${\beta }_{j}^{ini}$ is an initial estimate of the coefficients from the Ridge Regression and $\gamma$ is a positive constant for adjustment of the adaptive weights vector which was fine-tuned to 0.75 after testing values between 0.5 and 2 at increment levels of 0.25. A total of 26 out of 98 features with coefficient values of 0 were dropped from the model training process.

### Classifier design

Multiple machine learning classifiers were explored in this study, namely Logistic Regression, XGBoost, Light GBM, and Random Forest. However, the Random Forest model achieved the best performance based on AUPRC and AUROC as shown in Table 4 and thus was selected to predict the risk of a patient becoming part of the combined endpoint. A Random Forest is an ensemble machine learning algorithm that combines multiple decision trees to make predictions [14]. Hyperparameter tuning was performed with the objective of maximizing F1 score using the Tree-Structured Parzen Estimator algorithm [15]. F1 score is the harmonic mean of precision and recall. The tuned parameters included: maximum tree depth between 2 and 30, number of trees between 50 and 1000, minimum samples in leaf between one and six, minimum samples to split between two and eight and split criterion of either Gini impurity or entropy. For model explainability and interpretability, the importance of each feature was calculated based on their Shapley (SHAP) values [16]. SHAP was chosen for its ability to perform local explanation on both structured and unstructured data, which gave a better understanding of how individual features contributed to the overall predicted risk score on a case-by-case basis. This enables the model’s end users (Patient Navigators) to make informed decisions when developing tailored interventions for each patient in attempts to reduce their risk of readmission. Through the feature selection process, 72 features were selected for training of the final random forest model. To mitigate societal biases, a threshold optimizer from the Fairlearn toolkit was fitted and applied to the risk scores predicted by the random forest. The Fairlearn Threshold Optimizer helps find an optimal threshold for each sensitive group to minimize unfairness. All models were trained and tested on 38,581 cases using an 80/20% split of training and testing respectively (on a per surgical case basis). Note, this number differs from the 38,159 cases used for feature selection. This is because feature selection was implemented using adaptive lasso which is susceptible to outliers. Hence, the removal of 422 outlier cases from original 38,581 cases. However, random forest is not susceptible to outliers, thus, all cases were used, including the outliers. The random forest model was implemented through scikit-learn (https://scikit-learn.org/) and trained on 8-CPUs, each having 16 cores and 64 GB of RAM.

**Table 4:** Model performance

	Random forest	LGBM	XGBoost	Logistics regression
AUROC (95% CI)	0.738 (0.724, 0.754)	0.736 (0.723, 0.751)	0.734 (0.722, 0.751)	0.726 (0.711, 0.742)
AUPRC (95% CI)	0.406 (0.384, 0.433)	0.397 (0.368, 0.425)	0.397 (0.373, 0.428)	0.389 (0.367, 0.419)

### Bias detection and mitigation

**Fig. 1:**
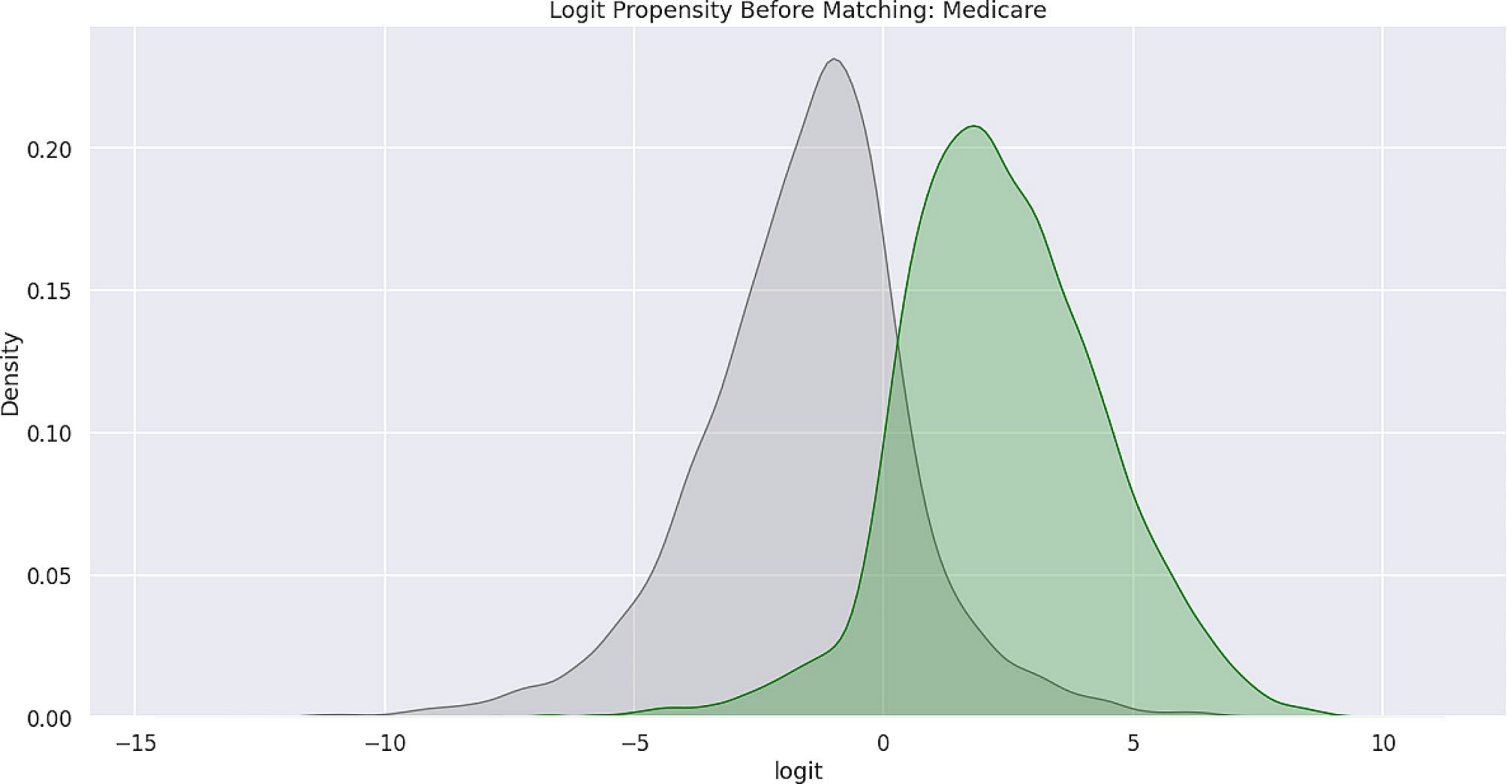
Logit propensity score distribution of Medicare status groups before matching

**Fig. 2:**
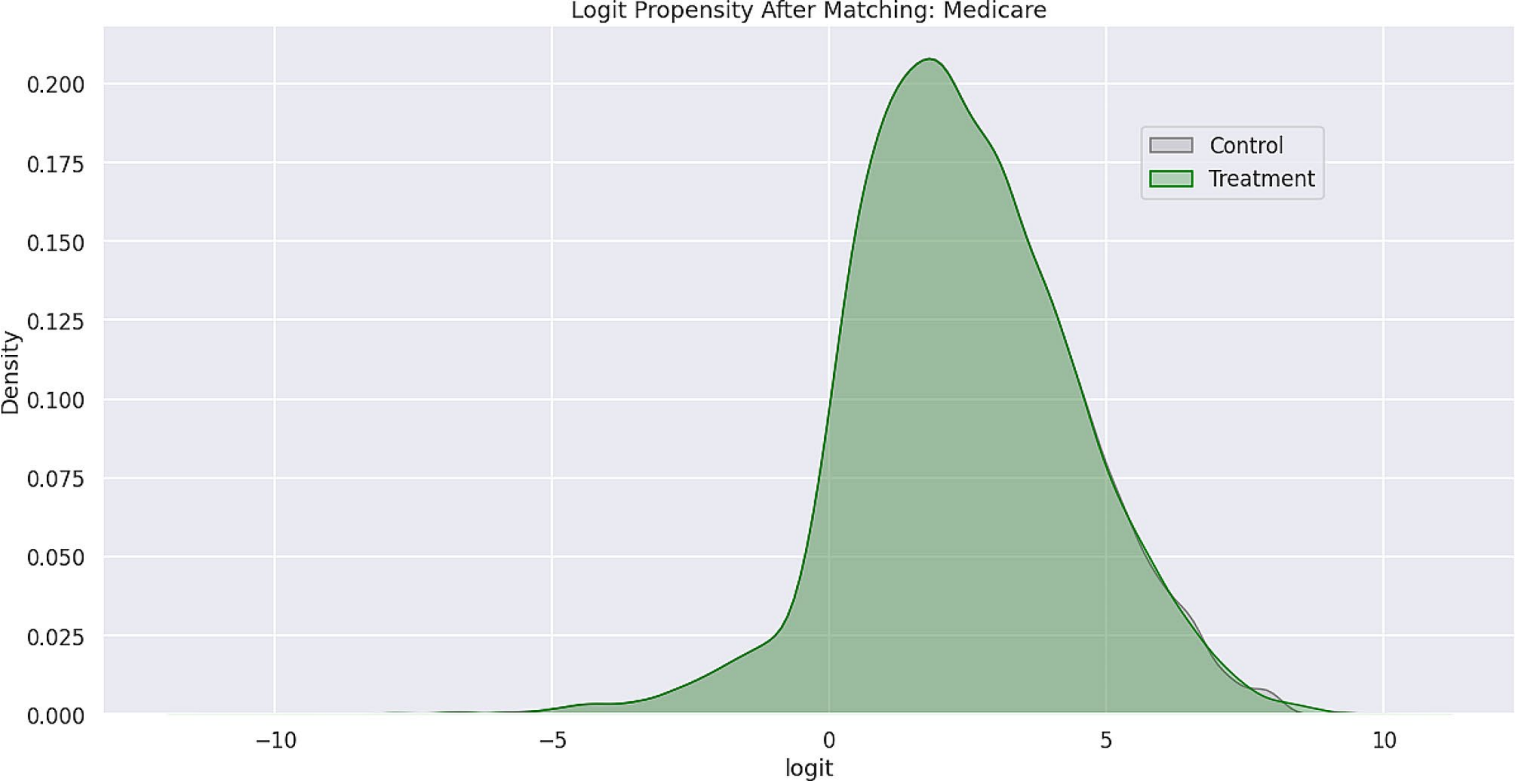
Logit propensity score distribution of Medicare status groups after matching

**Fig. 3:**
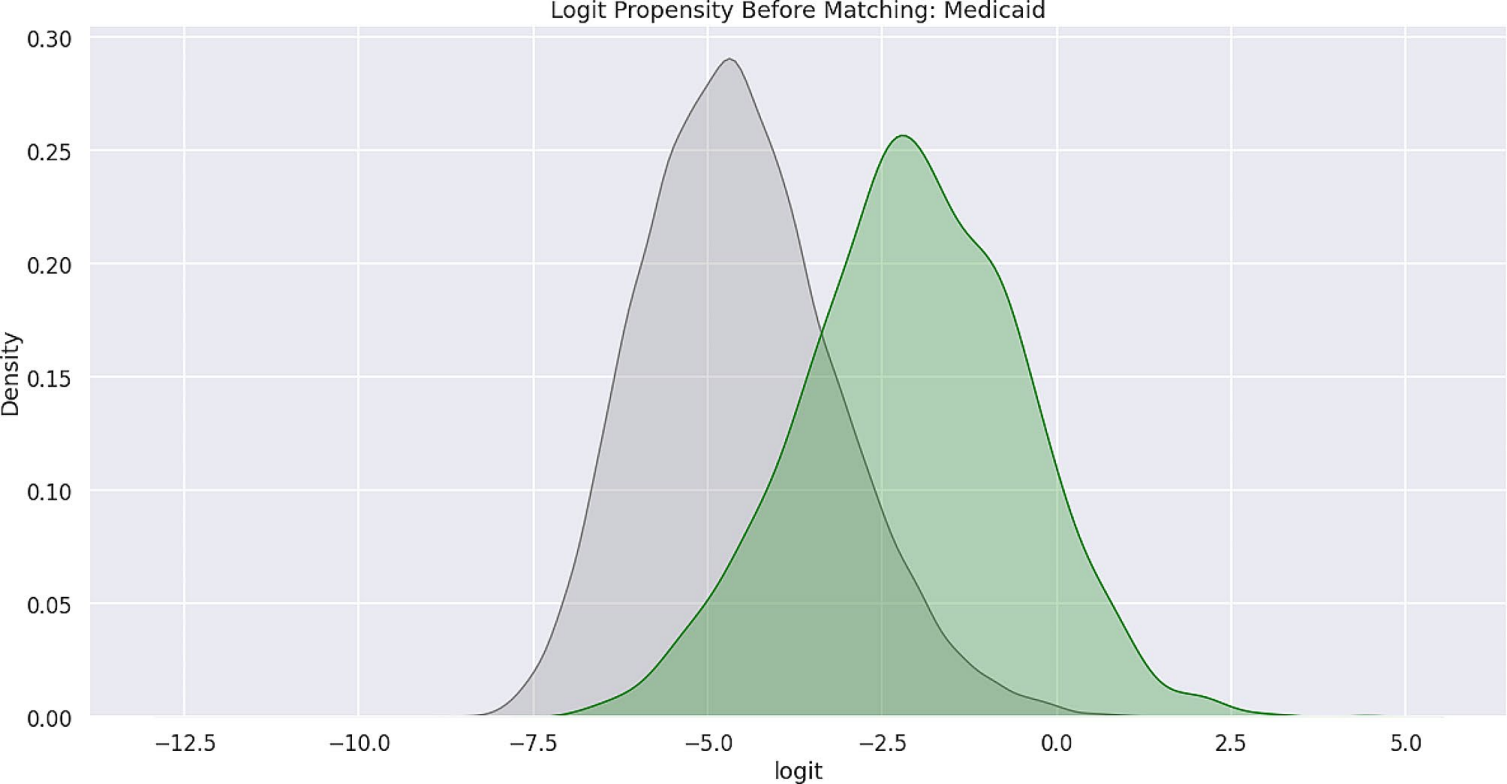
Logit propensity score distribution of Medicaid status groups before matching

**Fig. 4:**
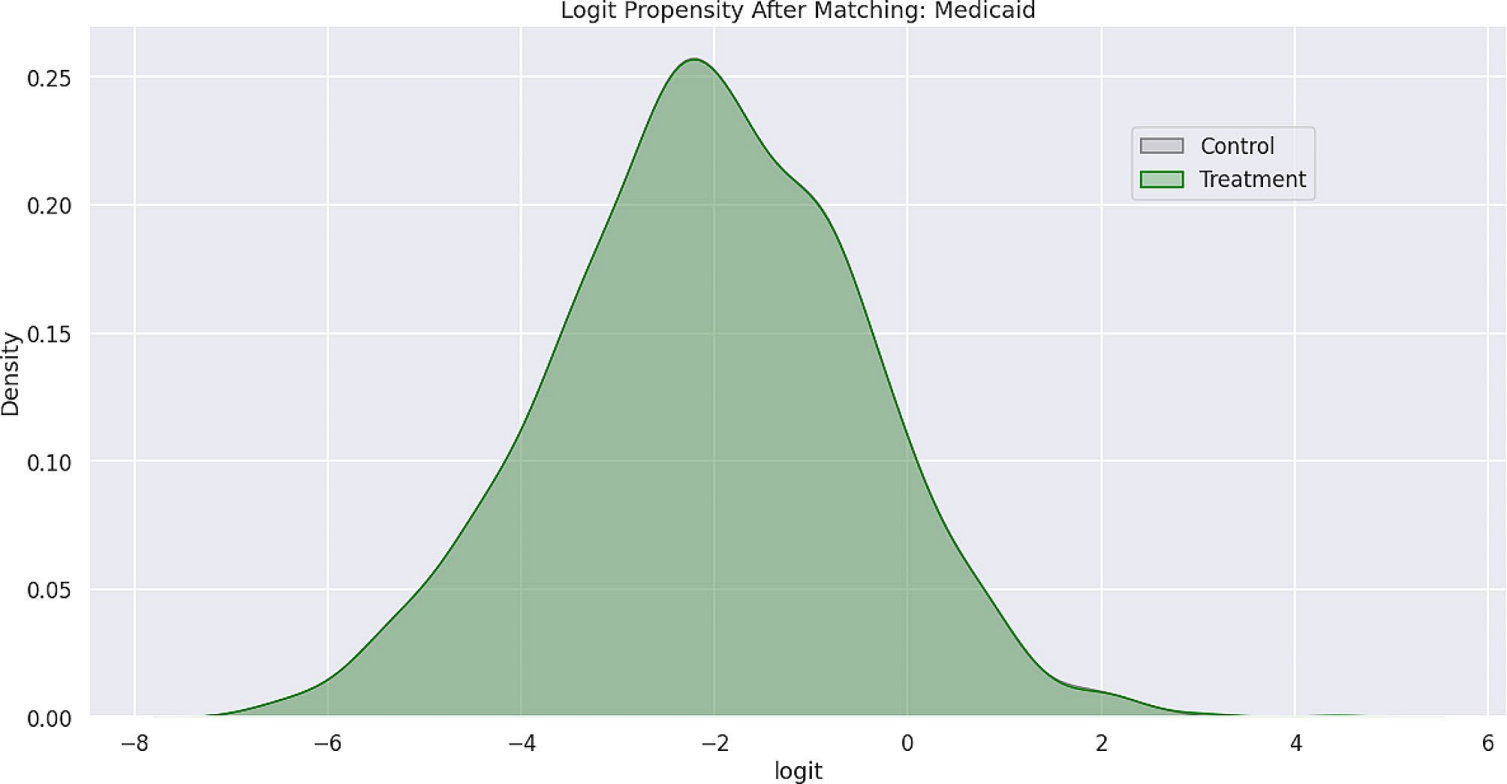
Logit propensity score distribution of Medicaid status groups after matching

**Fig. 5:**
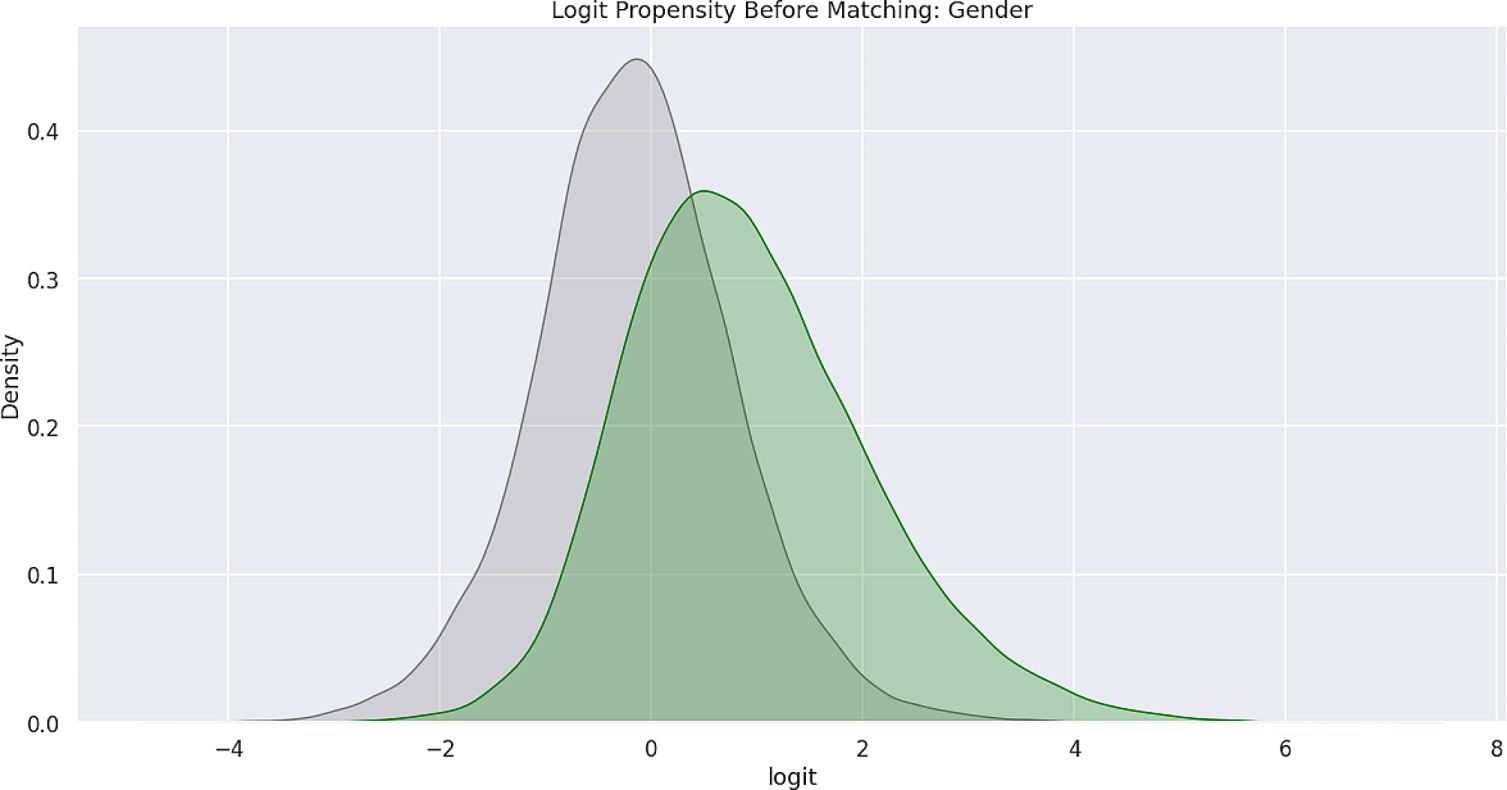
Logit propensity score distribution of gender groups before matching

**Fig. 6:**
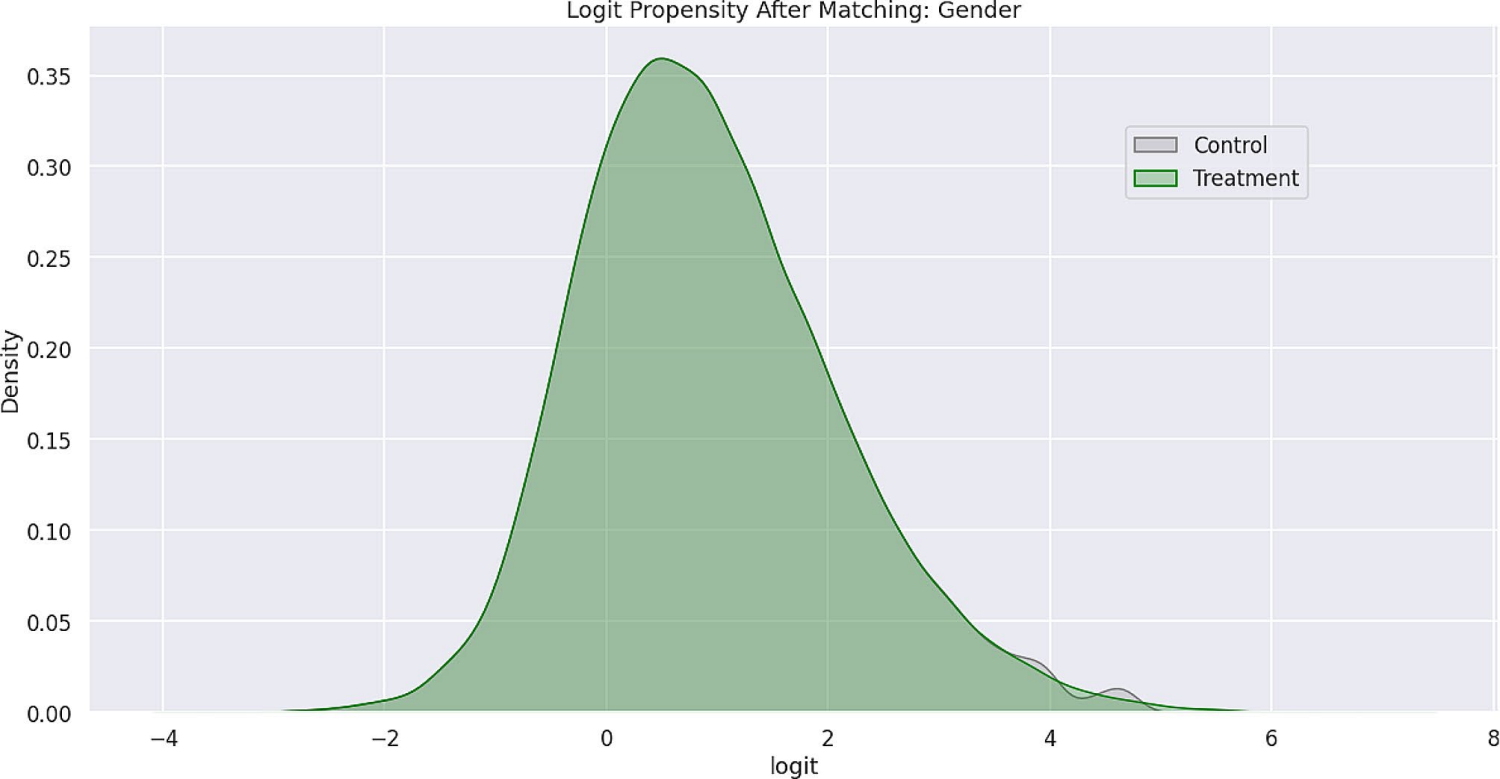
Logit propensity score distribution of gender groups after matching

Feature bias occurs when there is a negative impact on the learning process of a machine learning model due to the influence of certain sensitive features, which leads to less-than-optimal outcomes or predictions. Sensitive features, also known as protected attributes, refer to features that divide a population into groups that should be treated fairly and equally. These features have the potential to cause discrimination against certain subgroups, for example: sex, gender, family status, socio-economic classification, and other largely immutable characteristics. Within the scope of this study, three sensitive features were examined: Gender, Medicare Status, and Medicaid Status. Hypothesis testing was the first step carried out in the bias detection process. The overall goal was to utilize statistical inference to decide whether the predicted values for the combined endpoint between each sub-divided group differed significantly. There were significant differences between the predicted values within the subdivided groups as indicated by the p-values (< 0.001) results from the conducted Mann-Whitney U-Test. Subsequently, Propensity Score Matching (PSM) was utilized to delve further into the variations in predictions between the sub-divided groups to better understand the impact of each sensitive feature on the overall combined endpoint. The propensity score is the probability of treatment assignment conditioned on observed baseline characteristics. The propensity score allows one to design and analyze an observational (non-randomized) study so that it mimics some of the characteristics of a randomized controlled trial [17]. The matching technique utilized in this study for PSM was a 1:1 nearest neighbor match. The propensity score was estimated through logistic regression of the treatment variables against the covariates and matching was done without replacement. To assess the quality of matching, summary statistics and distribution plots between control and treatment groups were compared. Successful matching was ensured once it was confirmed that the Standardized Mean Differences (SMD) for the covariates were below 0.2. The SMD is the ratio of the difference in mean outcome between the groups (treatment and control) and their overall standard deviation. Before and after distributions of the propensity scores for each group are shown below in Figs. 1, 2, 3, 4, 5 and 6.

The average treatment effect of Gender, Medicare status and Medicaid status were 0.0145, 0.0327, 0.0308 respectively. In other words, the risk of a patient being a part of the combined endpoint increases when one is either Female or has an active Medicare or Medicaid status. Once bias is detected in a model, it is important to ensure group fairness. Group fairness is typically formalized by a set of three constraints (Demographic Parity, Equalized odds, Equal opportunity) on the behavior of the predictor called parity constraints (also called “criteria”). The parity constraint utilized in this study was equalized odds, which requires that the false positive rates and false negative rates be equal between all sub-divisions of a categorical sensitive feature. To create a fairer model, a threshold optimizer was fitted to the final predicted risk scores to produce more equal outcomes across these sensitive groups by generating different thresholds for each group. Threshold optimization is a post processing approach where group-specific thresholds are applied to the estimator to optimize the performance objective under a specific fairness constraint. The threshold optimizer module from the Fairlearn package takes in the estimator and sensitive features list as inputs and outputs a predictor that incorporates different threshold specific predictors specific to each sensitive feature and gives a final binary prediction.

**Fig. 7:**
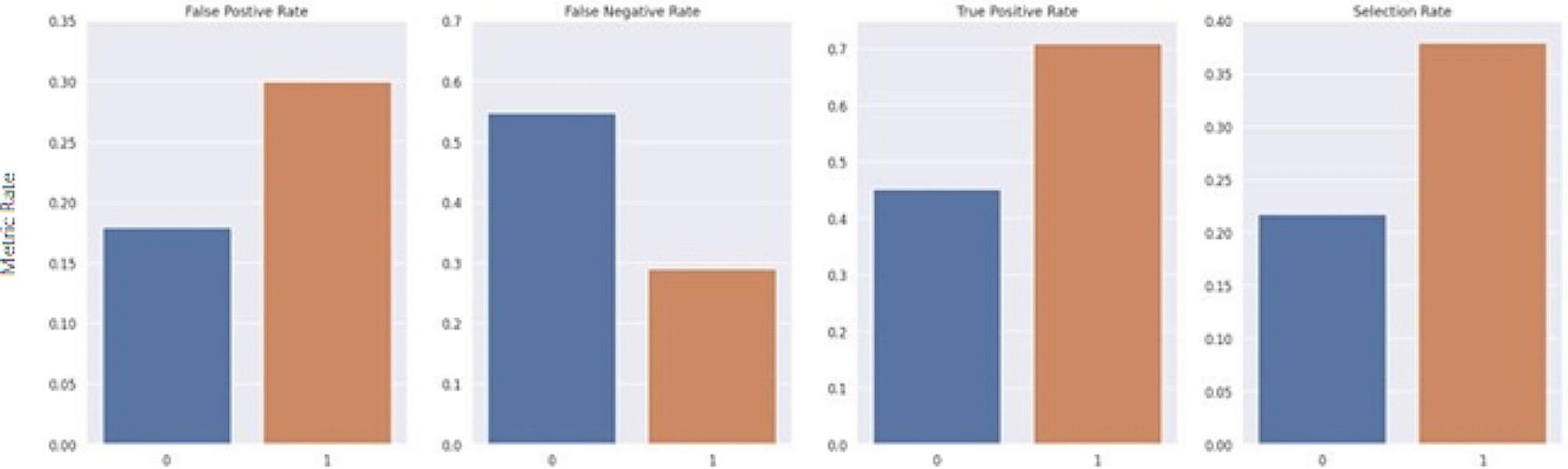
Fairness metric for gender groups before threshold optimization

**Fig. 8:**
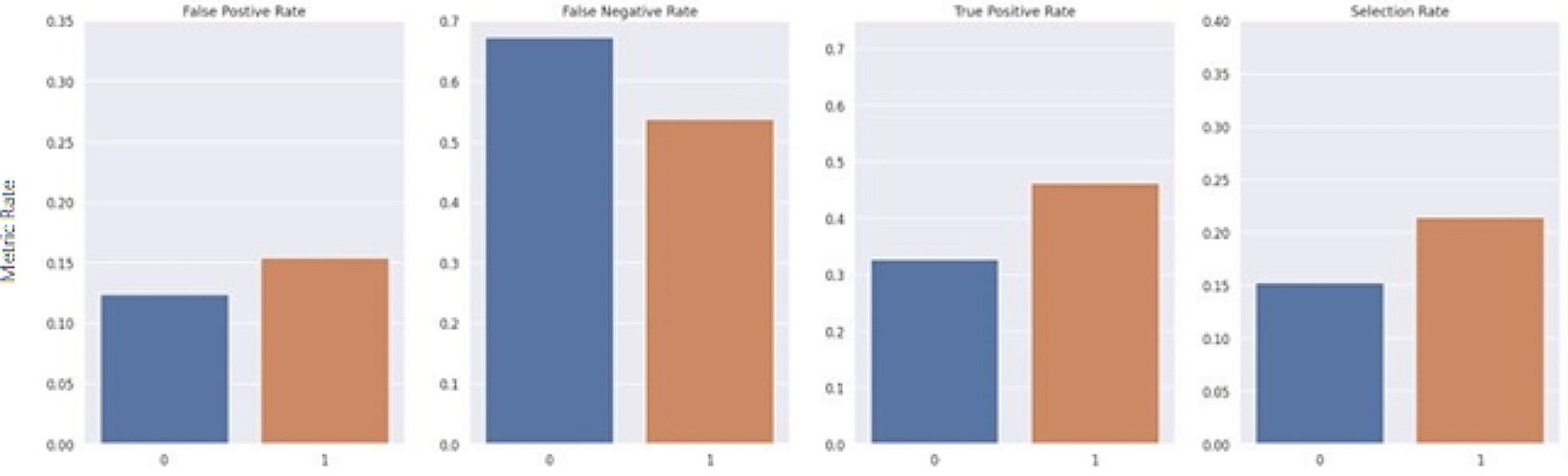
Fairness metric for gender groups after threshold optimization

**Fig. 9:**
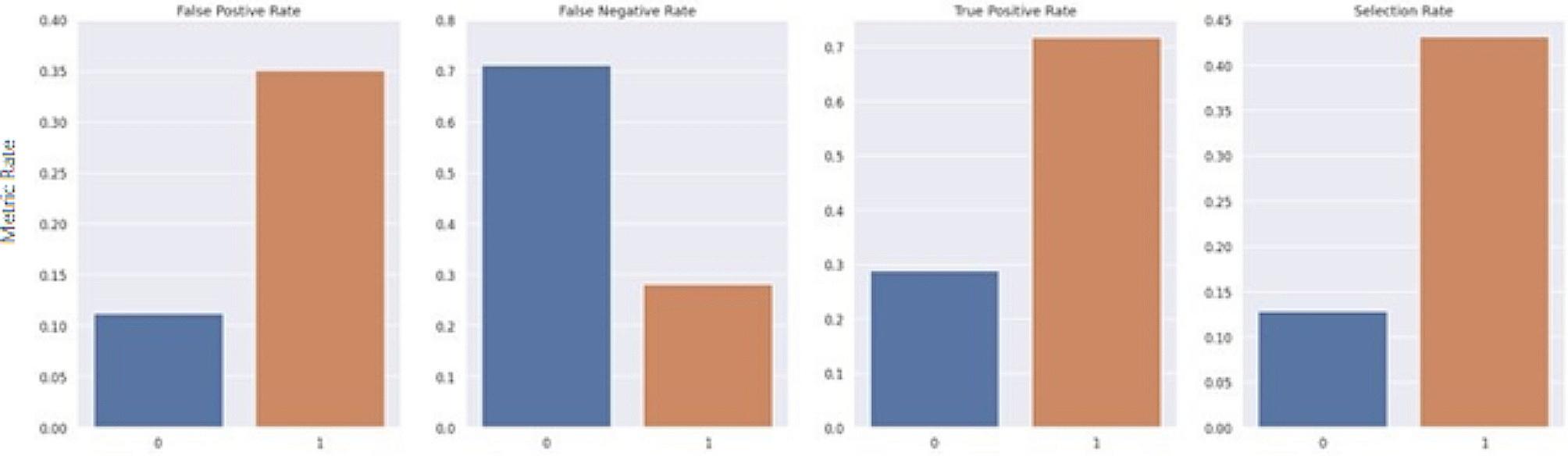
Fairness metric for Medicare status groups before threshold optimization

**Fig. 10:**
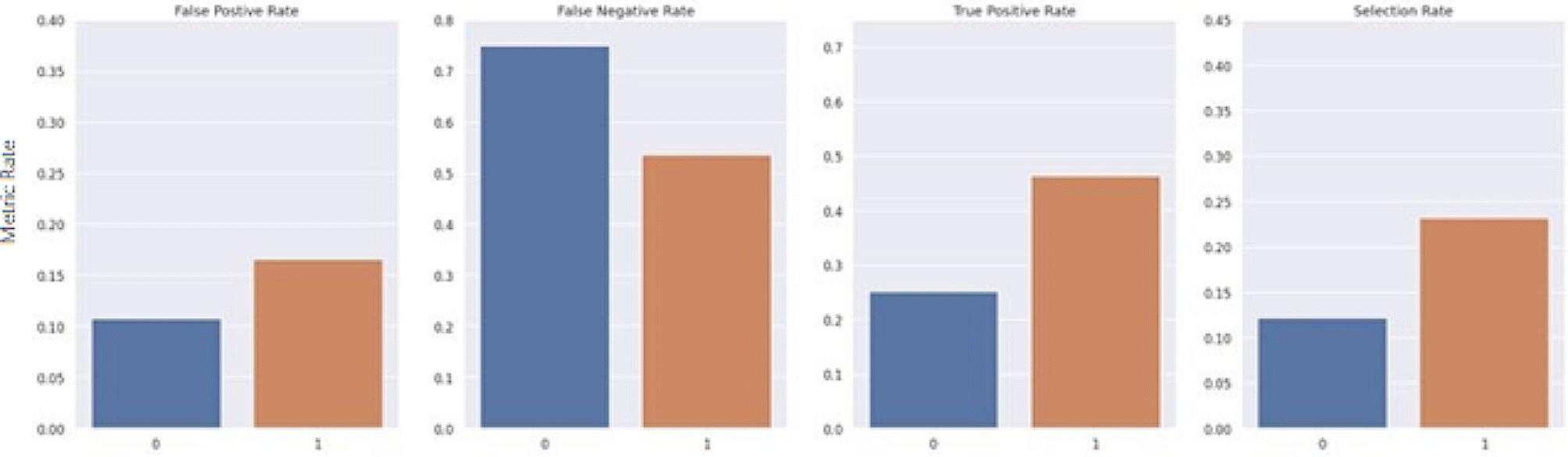
Fairness metric for Medicare status groups after threshold optimization

**Fig. 11:**
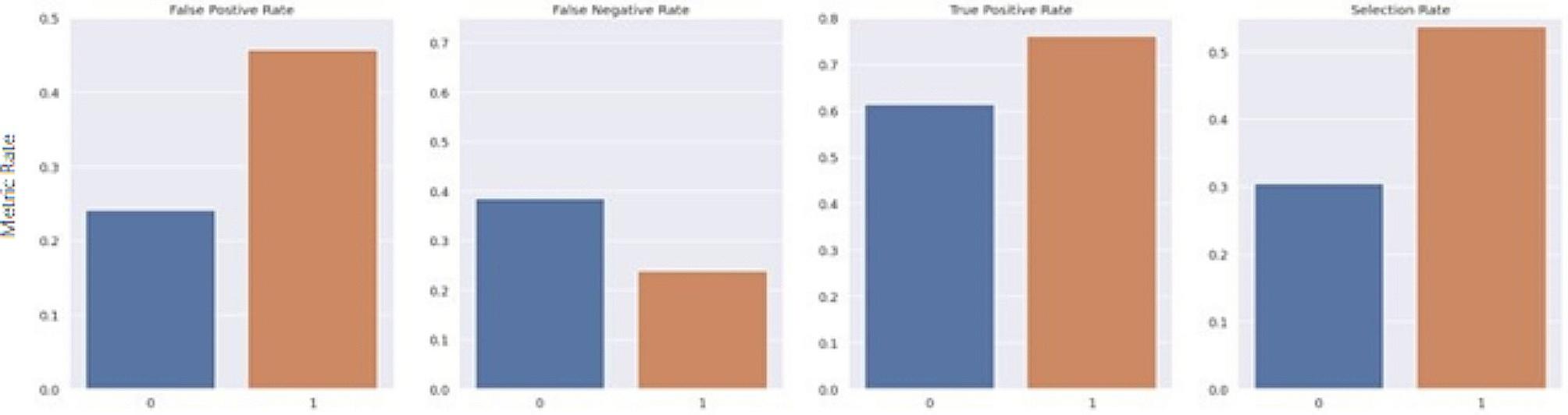
Fairness metric for Medicaid status groups before threshold optimization

**Fig. 12:**
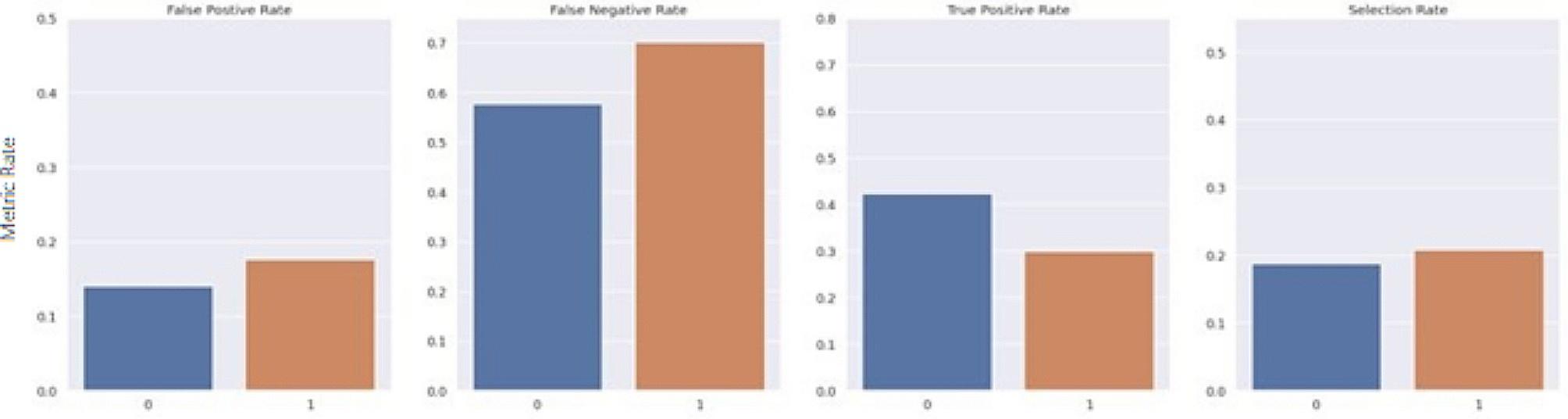
Fairness metric for Medicaid status groups after threshold optimization

The Fairlearn threshold optimizer is designed to generate ROC curves for each sensitive feature value at varying thresholds to select the optimal point by maximizing the objective from their overlapping region. The objective used in this study was the balanced accuracy score. The threshold optimizer yields two thresholds per sensitive group with an associated weight for each threshold. For the sake of consistency, it was important to keep predictions static and ensure that each case maintained the same predicted value each time the model was run. Thus, the randomization proposed in the original study was removed. For this study, rather than utilizing two thresholds per sensitive group, an extended version of the Fairlearn threshold optimizer was designed which utilizes a single threshold formulated by taking the weighted average of the thresholds. Furthermore, as a risk score is the desired outcome, this extended threshold optimizer applies a thresholding transformation to the original predicted score from the model, based on the newly formulated single threshold for each group. The transformation was computed by taking the ratio between the predicted risk score from the model and the formulated threshold. The range of the ratio is then confined to [0 ,1) by using a scaling function such that R_adj_ = x/(x + 1), where R_adj_ is the final adjusted risk score, and x is the ratio between the predicted risk score from the model and the formulated threshold for its associated group. Thus, if multiple patients have the same risk score but belong to different sensitive groups, the final adjusted risk score considers their corresponding threshold. Figures 7, 8, 9, 10, 11 and 12 show the difference in True Positive Rate and False Positive Rate for the different sensitive groups, before and after the extended threshold optimizer was applied, ensuring that the constraint of equalized odds for fairness had been met.

## Results

**Fig. 13:**
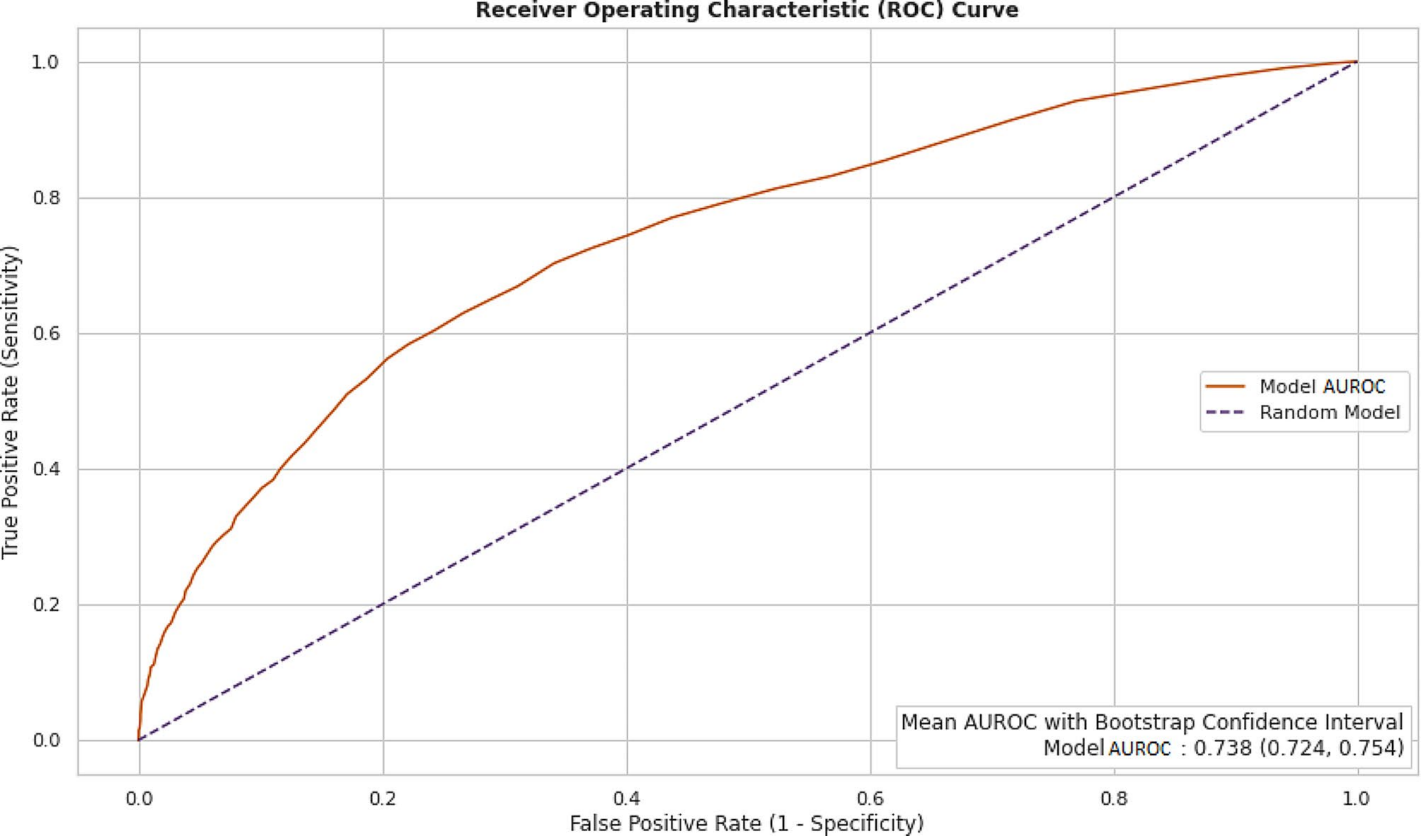
Performance metric of random forest model: receiver operating characteristic curve

**Fig. 14:**
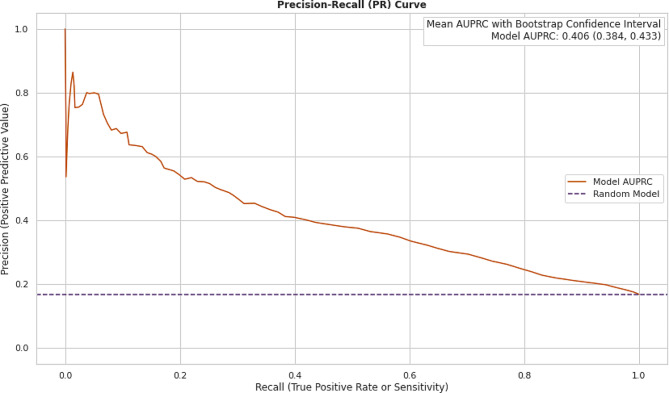
Performance metric of random forest model: precision-recall curve

The primary focus of evaluation was placed on the model’s ability to predict true positive cases (surgical case correctly predicted as part of the combined endpoint), rather than true negatives (surgical cases correctly predicted as not part of the combined endpoint). Thus, while training the model, significant attention was given to the area under precision recall curve (AUPRC) as it avoids the influence of true negatives. Figure 13, and Fig. 14 show the receiver operating characteristics curve (AUROC), and precision recall curve (AUPRC).

**Fig. 15:**
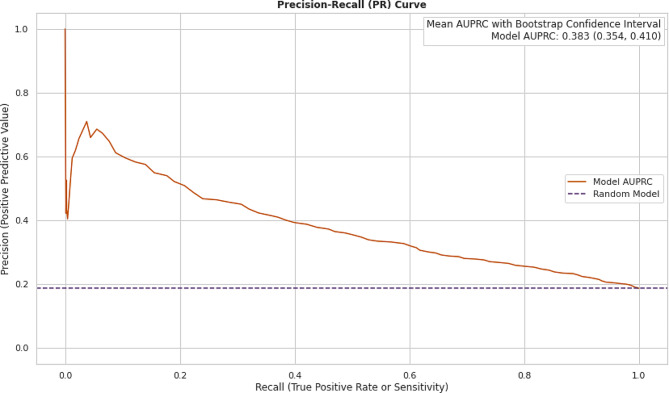
Performance metric of NLP model: precision-recall curve

**Fig. 16:**
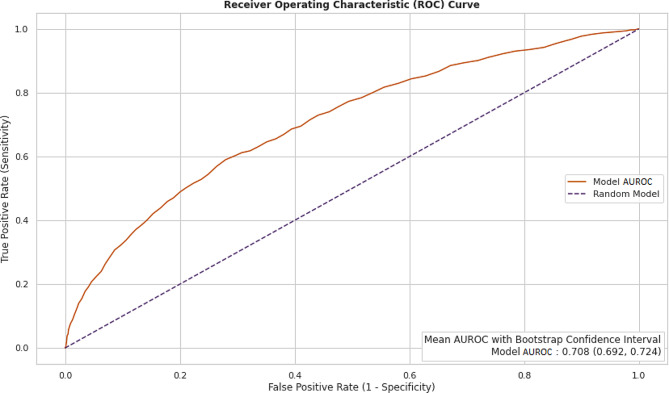
Performance metric of NLP model: receiver operating characteristic curve

The AUROC and AUPRC metrics for the BioClinicalBert NLP model trained on patient notes are shown in Figs. 15 and 16 below.

Table 5 details the final set of features used in the model and their corresponding global SHAP values in descending order of global importance. The top three predictive features were NLP risk score, relationship status, and age.

**Table 5:** SHAP importance

Predictor	SHAP importance	Description
NLP risk score	0.07953	The risk score given by NLP (BioClinicalBERT) model from notes
Relationship status	0.03664	If patient has significant other based on last known until case creation date
Age	0.0351	Patient age on the case creation date
Medicare status	0.03203	If the patient has active Medicare during case creation date
Anti-coagulants	0.0176	Ordered anticoagulants in the last 90 days until case creation date
Number of ED visits	0.01506	The number of ED visits the patient has had in the past two years from case creation date
BMI	0.01458	Last known BMI until case creation date
Charlson Score	0.01281	Charlson comorbidity index (CCI)—based on comorbid conditions
Max stay length	0.00954	The max days of length of stay the patient has had in the past two years from case creation date
Hematopoietic agents	0.00846	Ordered hematopoietic agents in the last 90 days until case creation date
Creatinine	0.00812	Most recent creatinine lab test value recorded in the last 2 years
Number of office visits	0.00722	The number of office visits patient has had in the last 90 days
Blood urea nitrogen	0.00722	Most recent blood urea nitrogen lab test value recorded in the last 2 years
Albumin	0.0069	Most recent albumin lab test value recorded in the last 2 years
Respiratory disorder	0.00631	If patient has been diagnosed with respiratory disorders within the last year of case creation date
White blood cell	0.00582	Most recent white blood cell count lab test value recorded in the last 2 years
Hemoglobin	0.00562	Most recent hemoglobin lab test value recorded in the last 2 years
Sex	0.00559	If patient sex is female or not
Prothrombin time	0.00552	Prothrombin time test for liver to measure how long it takes the blood sample to clot
Hematocrit	0.00551	Most recent Hematocrit lab test value recorded in the last 2 years
Total protein	0.00529	Most recent total protein content lab test value recorded in the last 2 years
A1C	0.00528	Most recent A1C content lab test value recorded in the last 2 years
Nervous system conditions	0.00521	If patient has been diagnosed with Nervous System Conditions within the last year of case creation date
Diuretics	0.00502	Ordered diuretics in the last 90 days until case creation date
Red blood cell	0.00465	Most recent red blood cell count lab test value recorded in the last 2 years
Alcohol use	0.00458	If the patient most recent social history indicates as an alcohol user
Anti-rheumatic	0.00454	Ordered anti-rheumatic in the last 90 days until case creation date
Illegal drug use	0.00402	If the patient most recent social history indicates as an illegal drug user
Number of surgeries	0.004	The number of prior surgeries the patient has had in the last year from the case creation date
Partial thromboplastin time	0.00365	Partial thromboplastin time tests are for the liver to measure how long it takes the blood sample to clot
Abdominal pain	0.00362	If patient has been diagnosed with abdominal pain within the last year of case creation date
Heart disorders	0.00346	If patient has been diagnosed with heart disorders within the last year of case creation date
Beta blockers	0.0033	Ordered beta blockers in the last 90 days until case creation date
Cardiac dysrhythmias	0.00306	If patient has been diagnosed with cardiac dysrhythmias within the last year of case creation date
Anti-diabetic	0.003	Ordered antidiabetic in the last 90 days until case creation date
Fall history	0.003	If patient has fallen within the last year of case creation date
Number of no show appts	0.00281	The number of no show appointments patient has had in the last 90 days from case creation date
Calcium blockers	0.00279	Ordered calcium blockers in the last 90 days until case creation date
Anti-emetics	0.0027	Ordered antiemetics in the last 90 days until case creation date
Corticosteroids	0.00261	Ordered corticosteroids in the last 90 days until case creation date
Vitamins	0.00259	Ordered vitamins in the last 90 days until case creation date
Fluid disorders	0.00247	If patient has been diagnosed with fluid & electrolyte disorders within the last year of case creation date
Pulmonary disease	0.00239	If patient has been diagnosed with chronic obstructive pulmonary disease & bronchiectasis within the last year of case creation date
Tobacco use	0.00239	If the patient most recent social history indicates as a tobacco user
Anti-parkinsonian	0.00234	Ordered antiparkinsonian in the last 90 days until case creation date
Analgesics-narcotics	0.00234	Ordered analgesics-narcotic in the last 90 days until case creation date
Urinary tract infections	0.00221	If patient has been diagnosed with urinary tract infections within the last year of case creation date
Upper respiratory infections	0.00212	If patient has been diagnosed with other upper respiratory infections within the last year of case creation date
Asthma	0.0021	If patient has been diagnosed with asthma within the last year of case creation date
Number of inpatient admissions	0.0021	The number of inpatient admissions the patient has had in the last year from case creation date
Medicaid status	0.00165	If the patient has active Medicaid during case creation date
Nausea & vomiting	0.00162	If patient has been diagnosed with nausea & vomiting within the last year of case creation date
Back problems	0.00156	If patient has been diagnosed with spondylosis intervertebral disc disorders & other back problems within the last year of case creation date
Anti-anginal	0.00155	Ordered antianginal agents in the last 90 days until case creation date
Anti-psychotics	0.00154	Ordered antipsychotics in the last 90 days until case creation date
Syncope	0.0013	If patient has been diagnosed with syncope within the last year of case creation date
Phlebitis, thrombophlebitis & thromboembolism	0.00124	If patient has been diagnosed with phlebitis, thrombophlebitis and thromboembolism within the last year of case creation date
Heart valve disorders	0.00118	If patient has been diagnosed with heart valve disorders within the last year of case creation date
Cognitive disorders	0.00116	If patient has been diagnosed with cognitive related disorders within the last year of case creation date
Nutritional disorders	0.00113	If patient has been diagnosed with metabolic or nutritional related disorders within the last year of case creation date
Gastrointestinal hemorrhage	0.0011	If patient has been diagnosed with gastrointestinal hemorrhage within the last year of case creation date
Fall risk	0.00107	The patient most recent fall risk index score obtained from flowsheets in the last year from the case creation date
Chest pain	0.00103	If had an ED visit with chest pain in last 730 days until case creation date
Substance related disorders	0.00098	If patient has been diagnosed with substance related disorders within the last year of case creation date
Non-infectious gastroenteritis	0.00095	If patient has been diagnosed with non-infectious gastroenteritis within the last year of case creation date
Acute cerebrovascular disease	0.00091	If patient has been diagnosed with acute cerebrovascular disease within the last year of case creation date
Respiratory failure	0.00071	If patient has been diagnosed with respiratory failure insufficiency arrest adult within the last year of case creation date
Imputed BMI	0.00054	Indicator stating whether imputation technique was utilized to obtain BMI value for patient
Acute myocardial infarction	0.00036	If patient has been diagnosed with acute myocardial infarction within the last year of case creation date
Protein in urine	0.00018	Most recent protein in urine content lab test value recorded in the last 2 years.
HIV Status	8e-05	If patient has been diagnosed with HIV Infection within the last year of case creation date
Spinal cord injury	0.0	If patient has been diagnosed with spinal cord injury within the last year of case creation date

## Discussion

This study proposes a machine learning based model to effectively predict the risk of a patient having an unplanned readmission, emergency visit, or discharge to a skilled nursing facility following total hip, shoulder, and knee joint replacement surgery. The study also utilizes threshold optimization to create fairer model outcomes for patients across sensitive societal groups. SHAP values were used to show model interpretability by quantifying the relative importance of each predictive feature in predicting a patient’s risk score.

In this study, patients without significant other were found to have a higher risk of combined endpoint. This is due to lack of care at home after the surgery. For such patients, discharge options such as home-care or discharge to assisted living facility or additional time to organize help at home can be provided to reduce the risk of combined endpoint. In agreement with previous study [18], our study observed that patients with high or low BMIs were at higher risk of combined endpoint. To reduce risk, obese or malnourished patients can be encouraged to follow diet management practices. Furthermore, surgery could be postponed until BMI is in the clinically acceptable range. Patients who have comorbidities are also at higher risk as represented by Charlson score being one of the significant risk factors. Usage of anti-coagulants was also a strong risk factor. It serves as an indicator to alert providers about potential existing blood clotting issues to consider during and after the surgery.

NLP Risk score is one of the most important features, it is imperative that this feature can be interpreted by providers who are reviewing it. NLP risk score indicates the risk of combined endpoint based on clinical notes. By using SHAP, the importance of each token of notes for each patient can be obtained. Using tokens alone to interpret the NLP risk score may not be completely feasible. SHAP combines group of tokens into chunks based on the interactions between groups. This results in chunks such as “patient declined steroid injection into her knee“, “patient has anemia, cancer”, along with their combined SHAP values. The chunks with higher SHAP values are sent to providers. This helps them understand what information in the clinical notes contribute towards increasing the risk score and also saves them tremendous amount of time needed to go through entire notes of the patient.

There are several limitations to the present study. First, it is possible that our study may not capture combined endpoint for patients who had surgery in our facility and were admitted or treated in a different institution within 30 days of surgery. Second, the risk of combined endpoint is predicted before surgery, hence, factors during surgery such as operative time, procedure complexity, and patient’s functional status post-surgery could play a role in the cause of readmission which were not considered for the study. Despite these limitations, our study can be used to preoperatively calculate the risk of combined endpoint effectively as demonstrated by an AUROC of 0.738 (95% confidence interval, 0.724 to 0.754) and an AUPRC of 0.406 (95% confidence interval, 0.384 to 0.433). Thus, aiding provider’s decision-making which optimizes the patient outcomes.

## Conclusion

The model’s ability to identify high-risk patients could prove valuable for further efforts aiming to provide clinical decision support for clinicians to optimize individual patient care. High-risk patients may benefit from additional preventative efforts, such as pre-surgery education about blood sugar management to diabetes patients or pain management discussions, to minimize the risk of complications following surgery. Clinicians may leverage this tool to more accurately convey to patients what factors and behaviors they can modify pre-surgery to ensure the best surgical outcome. Overall, the model in this study serves as a robust clinical decision support tool which aids in the identification of high-risk patients that will need additional clinical intervention to prevent readmissions and ED visits and ensure effective recovery.

## Data Availability

No datasets were generated or analysed during the current study.
